# Reflections on the Global Burden of Disease 2010 Estimates

**DOI:** 10.1371/journal.pmed.1001477

**Published:** 2013-07-02

**Authors:** Peter Byass, Maximilian de Courten, Wendy J. Graham, Lucie Laflamme, Affette McCaw-Binns, Osman A. Sankoh, Stephen M. Tollman, Basia Zaba

**Affiliations:** 1 Umeå Centre for Global Health Research, Umeå University, Umeå, Sweden; 2 School of Public Health, Faculty of Health Sciences, University of the Witwatersrand, Johannesburg, South Africa; 3 Copenhagen School of Global Health, University of Copenhagen, Copenhagen, Denmark; 4 Institute of Applied Health Sciences, School of Medicine and Dentistry, University of Aberdeen, Aberdeen, United Kingdom; 5 Department of Public Health Sciences, Karolinska Institutet, Stockholm, Sweden; 6 Department of Community Health and Psychiatry, University of the West Indies, Mona, Kingston, Jamaica; 7 INDEPTH Network, Accra, Ghana; 8 Hanoi Medical University, Hanoi, Viet Nam; 9 London School of Hygiene and Tropical Medicine, London, United Kingdom

## Abstract

Peter Byass and colleagues raise questions about the recent, high-profile Global Burden of Disease estimates.

*Please see later in the article for the Editors' Summary*

**Summary Points**

- Health data include many gaps, particularly relating to poorer areas of the world, so complex estimation techniques are needed to get overall global pictures.
- Estimates of population health, however, carry their own uncertainties and may be flawed in some instances.
- Here we present a range of reflections on the Global Burden of Disease 2010 estimates, highlighting their strengths as well as challenges for potential users.
- In the long term, there can be no substitute for properly counting and accounting for all the world's citizens, so that complex estimation techniques are not needed.

The Institute for Health Metrics and Evaluation (IHME) and its partners recently completed what is probably the largest ever exercise undertaken in epidemiological modelling, the Global Burden of Disease 2010 (GBD-2010) estimates [1]. These estimates attempt to characterise loss of health from disease and injury, including the effects of some major risk factors, on a global basis. They will find widespread use in coming years and influence developments in global health. However, it is important to realise that “estimates are estimates, and not measurements"; they may perform better in some respects than others [2]. Here, as a group of independent experts, we comment on some of the major issues raised by this important work, while noting that it is impossible to cover all the wealth of detail involved in any critique. We take collective responsibility for these views, though many specific points come from individual specialists among the authors.

## What Are the Underlying Data and Uncertainties for GBD-2010?

**Figure 1:**
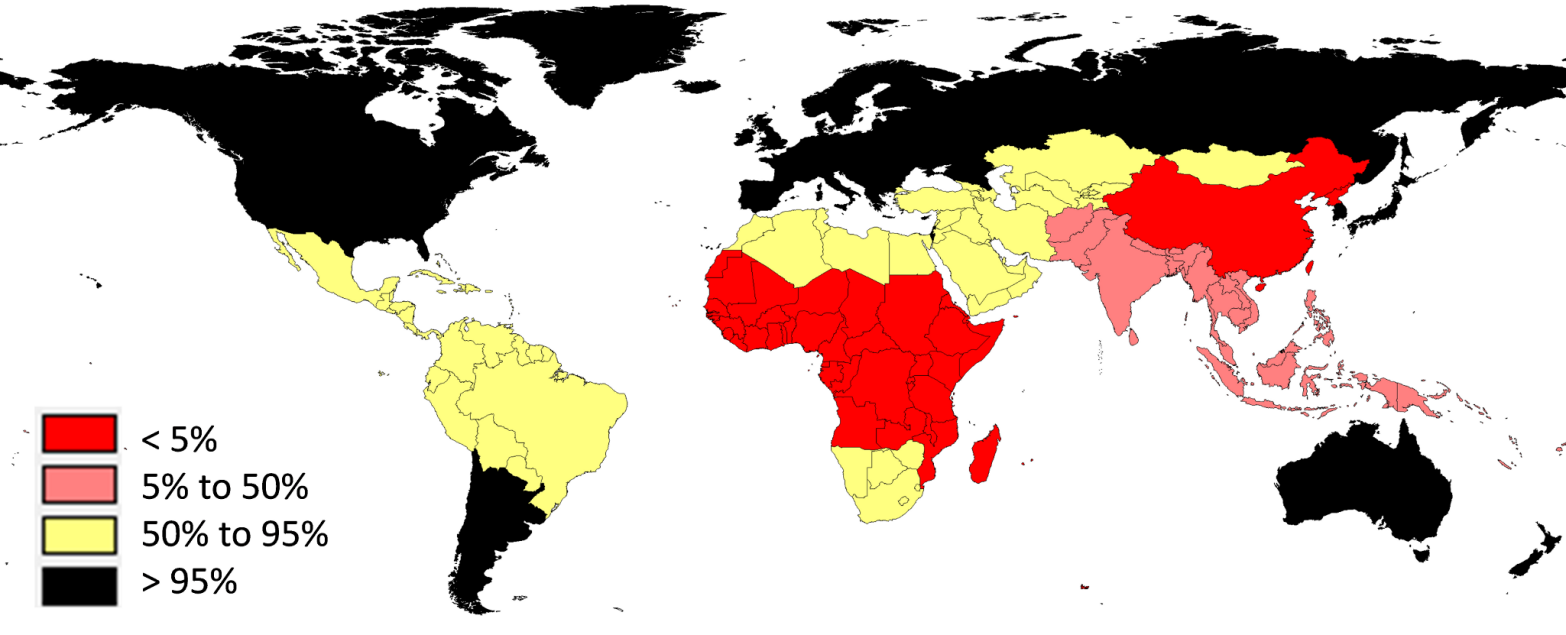
Proportions of deaths covered by vital registration, shown by GBD-2010 regions. Data from [20]; unregistered deaths from the 2010 Haiti earthquake excluded.

There is sparse description of the source database compiled for GBD-2010, and it is not publicly available. The most detailed overview of the underlying data comes from a single quote: “We have included almost 800 million deaths from 1950 to 2010, and the data come from different sources. The goal was to incorporate ‘all the available data’" [3]. As the GBD-2010 group acknowledges, these data in fact correspond to only around 30% of global deaths over the whole period, and are a mixture of survey data, sample registration, and vital registration [4]. According to World Health Organization (WHO) data, vital registration coverage has risen in recent years to around 40% of global deaths, but with a very unequal global distribution, as shown by GBD-2010 region in Figure 1. Consequently, the majority of the deaths in the GBD-2010 database must have come from areas with fairly complete vital registration, though no doubt the sophisticated GBD-2010 modelling adjusts for this bias as far as possible. However, more than 30% of the world's population live in regions where less than 5% of all deaths are registered—a critical ongoing concern for understanding global health [5]. Since GBD-2010 included all possible data, it is difficult to determine the external validity of the findings beyond the available data, or to establish the overall validity of the estimates.

**Figure 2:**
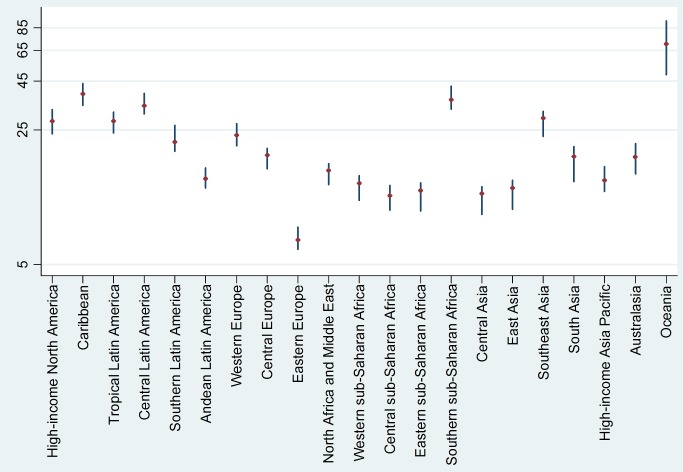
Diabetes mortality rates per 100,000 (with 95% uncertainty intervals) by GBD-2010 region for 2010. Data from [21].

A good development across all the GBD-2010 work is the 95% uncertainty intervals calculated around the results. However, given the complex nature of the modelling for the point estimates, these intervals are also complex. In Figure 2, as an example, the mortality rates for diabetes are shown, by GBD-2010 region, with their uncertainty intervals. Interestingly, in regions where there are only scant data on diabetes mortality, such as in sub-Saharan Africa, the intervals are not appreciably wider than in other regions with much more comprehensive data, suggesting that the uncertainty intervals reflect more of the internalities of the modelling rather than the quality and quantity of source data. Understanding the construction and interpretation of this plethora of GBD-2010 uncertainty intervals remains an ongoing challenge.

## Building on Previous Global Burden of Disease Work

The GBD-2010 team, at the London launch event in December 2012, emphasised that GBD-2010 estimates supersede previous estimates for earlier periods and differ in some respects. Much has rightly been made of substantial global increases in the numbers of elderly people and the impact of non-communicable disease (NCD) in recent years. Nevertheless, given the inevitability of NCD deaths at the elder extremes of populations, isolating the public health importance of premature NCD morbidity and mortality also remains critical.

The Ghana National Health Planning Unit (NHPU), in the late 1970s, developed a method whereby the health impact of different disease problems could be estimated quantitatively [6]. This method estimated loss of healthy life due to death, disablement, and illness for about 50 causes, which were then ranked. This approach was similar methodologically to Murray and Lopez's subsequent first attempt to assess healthy life lost due to different diseases on a global basis [7].

It is interesting to compare these earlier disease rankings for Ghana with GBD-2010 estimates for 1990 and 2010, as well as the WHO Burden of Disease findings for 2004. Table 1 compares rankings for the top fifteen GBD-2010 causes of lost years of healthy life—disability-adjusted life years (DALYs)—for Ghana in 2010 with the previous estimates [6],[8]. Many conditions maintain a relatively stable ranking across all four estimates. There are some stark differences with obvious explanations, such as the burgeoning burden of HIV/AIDS and the remarkable success of measles vaccination programmes. However, rankings for anaemia and diarrhoea vary widely, and sickle cell disorders are ranked substantially higher in the 1980 NHPU estimates than in the GBD-2010 estimates, even though the prevalence of this genetically determined condition in West Africa cannot have changed markedly. These latter examples illustrate the difficulties of translating various estimates into policy, being unsure whether differences reflect changes in methods and data, or real transitions.

**Table 1:** Top fifteen ranked, and selected other, causes of disability-adjusted life years for Ghana from GBD-2010 for 2010, compared with rankings from NHPU for 1980, GBD-2010 for 1990, and WHO Burden of Disease for 2004.

Cause of DALYs	NHPU 1980^a^	GBD-2010 for 1990^b^	WHO Burden of Disease 2004^c^	GBD-2010 for 2010^b^
Malaria	1	1	1	1
HIV/AIDS	—	7	2	2
Lower respiratory infections	2	2	4	3
Neonatal sepsis	22^d^	5	5	4
Preterm birth complications	6	6	7	5
Protein-energy malnutrition	5	8	20	6
Neonatal encephalopathy	7	9	6	7
Iron-deficiency anaemia	34^e^	11	23	8
Stroke	11	12	13	9
Meningitis	19	10	35	10
Diarrhoeal diseases	9	3	3	11
Road injury	8^f^	16	13	12
Ischaemic heart disease	31	17	16	13
Major depressive disorder	42	15	12	14
Epilepsy	—	19	25	15
Tuberculosis	10	13	9	17
Measles	3	4	44	36
Sickle cell disorders	4	20	-	24
Maternal causes	15	18	8	21

a Data from [6].

b Data from [22].

c Data from [23].

d Did not include all infections.

e Hookworm anaemia.

f Includes all causes of injury.

## Biomedical Plausibility

GBD-2010 has fitted all diseases and injuries into a 291-cause hierarchy that incorporates 235 causes of death [9]. This approach is inevitably a simplification of what happens in real life, whilst many individual episodes of disease and causes of death may not in reality be diagnosed and documented with sufficient precision to be categorised even within this framework. Some region-age-sex disease categories may not therefore be attributed with certainty. This is reflected in GBD-2010 by over 56,000 deaths in 2010 estimated to be in region-age-sex categories where the lower bound of the 95% uncertainty interval is zero, presumably indicating that there were possibly no such cases. Eighty-six of these zero-bound categories each related to more than 100 possible cases of otitis media, diphtheria, whooping cough, varicella, schistosomiasis, and other haemoglobinopathies.

Some potentially important sub-categories of disease have not been included in GBD-2010. For example, there is no distinction made between infections with different species of malaria parasite, even though there are important geographic and clinical differences between *Plasmodium falciparum* and *P. vivax* disease. Some causes of disease have been aetiologically defined (for example, shigellosis) even though—particularly when using data sources such as verbal autopsy (VA, where cause of death is determined by interviewing witnesses)—these may be attributed on a presumptive basis from symptoms. Several pathogen-specific categories, for example, various aetiologies of respiratory infections, seem to have relative estimates that differ from established knowledge, and this remains an area for further discussion. In addition, the relatively short-term effects of some new vaccines may shift disease patterns faster than would otherwise be expected, which may be difficult to model. Conversely, there may be dangers in relying too heavily on GBD-2010 estimates as a basis for major policy decisions, such as the introduction of new vaccines.

The consequences of the HIV/AIDS pandemic are a major difficulty for global estimates. Morbidity and mortality data are commonly not linked to individual evidence on HIV status, other than in specific contexts such as the ALPHA Network [10], and so modelling the effects of HIV on overall estimates can be misleading.

## The Dynamics of Maternal Mortality

Evidence of the dynamic nature of maternal mortality goes back centuries in some parts of the world. However, changes in the magnitude, causes, broader determinants, and risk groups of maternal mortality are only just emerging at the global level, since improved data sources and analytic methods are recent developments for many low-income countries. These changes in maternal mortality reflect the benefits of interventions such as family planning and emergency obstetric care, as well as the neglect of emerging causes of disease such as NCDs, and have major programmatic implications for the future. Exercises like GBD-2010 can undoubtedly help to both illuminate shifts and inform programme responsiveness. To realise this opportunity requires not only deeper probing of the data, but, crucially, engagement and empowerment of stakeholders in low-income countries to move the evidence into action.

GBD-2010 revitalises the metric of age-specific mortality rates for women of reproductive age, rather than considering maternal deaths in isolation, and this is welcome. However, since pregnancy is not included among the GBD-2010 risk factors [11], the GBD-2010 estimates do not address indirect maternal deaths, and hence do not contribute to understanding interactions between pregnancy and HIV/AIDS in terms of mortality [12]. GBD-2010 can help show where there is improved access to quality maternity services (evident from declines in total deaths) and better use of family planning (faster declines in some age-specific mortality rates). However, family planning use also changes the natural composition of the cohort of childbearing women, including those at higher risk. For example, in Jamaica between 1981 and 2011, absolute births declined faster (−33.7%) than maternal deaths (−31.7%), resulting in a stagnating maternal mortality ratio. More support needs to be available for countries to work with GBD-2010 estimates and better understand the consequences of the dynamic burden of maternal mortality.

## Why Are Injuries Important in GBD-2010?

Apart from making important contributions to morbidity and mortality worldwide, injuries as a health problem have special characteristics and are heterogeneous. Some mechanisms of injury, such as falls, occur more commonly with increasing proportions of elderly people in populations. Others are more random—as indicated by the profile of pedestrians injured by motor vehicles. Patterns of some types of injury are influenced by technological developments—increased speed, mechanisation, and industrialisation—while others may be socio-medically determined—such as suicide. All such factors make estimates of injuries complex.

Risk factors and causes of injuries are generally not well captured by the indicators utilised in GBD-2010, which focus on health risk behaviours and particular environmental exposures. Road traffic injuries, for instance, cannot be easily predicted or understood in light of those factors. Although alcohol is an acknowledged risk factor for road traffic crashes, motorisation, speed, and mixed traffic are far more important.

Injuries are just as much a challenge for the health sector as other GBD-2010 outcomes, though the health sector seldom considers determinants of injury as its responsibility [13]. From the DALYs presented, addressing the consequences of injuries in a timely manner (pre-hospital and hospital care) and providing rehabilitation to victims are imperatives. Not doing these brings the expense and burden of increased—and preventable—disability.

## Continuing Controversies in Malaria

Ahead of GBD-2010, IHME published separate estimates for the global burden of malaria [14]. Most controversially these suggested a much higher burden of malaria among adults than most experts expected. GBD-2010 to a large extent repeats the earlier IHME estimates for malaria, though there are some differences—and it is important to realise that this is not an independent confirmation of the earlier results. The continuing debate on the reality of the global malaria burden is, however, important [15].

There is agreement among malaria scientists on the lack of sufficient information on malaria-related deaths, especially in endemic countries where most of the deaths take place at home and many of the dead are buried without having being seen by a qualified healthcare provider. These circumstances make it hard to account for every death and even more difficult to determine cause of death. Irrespective of this unfortunate situation, efforts geared towards improving data availability seem to have been very limited relative to the extent of the problem.

While good health information systems might measure malaria-related mortality, only a small minority of malaria deaths occur within well-functioning healthcare systems, especially in sub-Saharan Africa. IHME's estimates of adult malaria mortality were partly based on a global VA series of more than 12,000 deaths at referral facilities, but which contained only 100 adult malaria deaths, mostly from India [16]. Alternative population-based approaches such as INDEPTH's health and demographic surveillance systems are important for capturing—using VA—deaths that occur outside the healthcare system [17]. Despite possible limitations of VA for detecting malaria deaths, it is currently the only option for most cases.

## Ways Forward

While GBD-2010 is undoubtedly a massive achievement for global health, our discussion above also reveals continuing concerns. WHO Director General Dr. Margaret Chan observed “We must not forget that the real need is to close the data gaps, especially in low-income and middle-income countries, so that we no longer have to rely heavily on statistical modelling for data on disease burden. We know that this will require stronger country health information systems, such as registration of births and deaths" [18]. A subsequent expert consultation convened by WHO in Geneva in February 2013 called for greater capacity investment in country-based estimates and standards of transparency [19]. As the more detailed material from GBD-2010 continues to be released, and possibly superseded by future revisions, there will also be continuing questions about the validity, reliability, transparency, and plausibility of the GBD-2010 findings. Planners and policy-makers, in particular, need to come to an understanding of how much reliance they should reasonably place on these estimates, especially in data-sparse countries.

## Abbreviations

DALY: disability-adjusted life year
GBD-2010: Global Burden of Disease 2010
IHME: Institute for Health Metrics and Evaluation
NCD: non-communicable disease
NHPU: National Health Planning Unit
VA: verbal autopsy
WHO: World Health Organization
